# Robotic Versus Sternotomy, Thoracotomy and Video-Thoracoscopy Approaches for Thymoma Resection: A Comparative Analysis of Short-Term Results

**DOI:** 10.3390/jpm15010034

**Published:** 2025-01-17

**Authors:** Beatrice Trabalza Marinucci, Matteo Tiracorrendo, Camilla Vanni, Fabiana Messa, Giorgia Piccioni, Alessandra Siciliani, Silvia Fiorelli, Mohsen Ibrahim, Erino A. Rendina, Antonio D’Andrilli

**Affiliations:** 1 Department of Thoracic Surgery, Sant’Andrea, Hospital, Sapienza University, 00189 Rome, Italy; beatrice.trabalzamarinucci@uniroma1.it (B.T.M.); matteo.tiracorrendo@ospedalesantandrea.it (M.T.); camilla.vanni@ospedalesantandrea.it (C.V.); fabiana.messa@uniroma1.it (F.M.); giorgia.piccioni@uniroma1.it (G.P.); alessandra.siciliani@ospedalesantandrea.it (A.S.); mohsen.ibrahim@uniroma1.it (M.I.); erinoangelo.rendina@uniroma1.it (E.A.R.); 2 Department of Anesthesiology, Sant’Andrea, Hospital, Sapienza University, 00189 Rome, Italy; silvia.fiorelli@uniroma1.it

**Keywords:** robotic-assisted thoracic surgery, thymoma, thymectomy, sternotomy, thoracotomy, VATS

## Abstract

**OBJECTIVE**. The optimal surgical approach for thymoma resection is still an object of debate. The increasing experience in robotic-assisted thoracic surgery (RATS) has led to the progressive affirmation of this technique as a valid alternative to Sternotomy, Thoracotomy and Video-Assisted Thoracic Surgery (VATS) in this setting. The present study aims to compare the post-operative and short-term results of RATS Thymectomy for thymoma with those of other main surgical approaches (sternotomy, thoracotomy and VATS) from a high-volume single center. **METHODS**. Between May 2021 and September 2023, 40 consecutive patients underwent RATS Thymectomy for stage I to limited-stage III thymoma in our center. Three homogenous groups of patients who received thymoma resection through main alternative approaches (sternotomy, thoracotomy, VATS) over the last 5 years, were identified in order to perform a comparative analysis. Data including surgery duration, associated resections, conversion rate, overall morbidity, tumor size, radicality of resection, post-operative pain, length of hospital stay and cosmetic results were retrospectively collected and compared between the RATS and each control group. **RESULTS.** Mean tumor size was higher in the sternotomy group, but not significantly. The mean operative time of RATS interventions was significantly lower than that of sternotomy and VATS. It was significantly shorter compared to thoracotomy if excluding docking-undocking time. A higher rate of associated adjacent structures resection was reported in the sternotomy group (*p* = 0.005). Conversion rate was significantly higher in the VATS group (*p* = 0.026) compared to RATS. Post-operative pain at 24 and 48 h was significantly lower in the RATS group compared to the others. Improved cosmetics results were reported after RATS compared to sternotomy (*p* = 0.0001) and thoracotomy (*p* = 0.001) groups, with a trend towards better results compared to VATS (*p* = 0.05). Length of hospital stay was shorter in the RATS group with a significant difference vs. the sternotomy group (*p* < 0.001). **CONCLUSIONS.** These results from a single center confirm the safety and efficacy of RATS for the treatment of limited stage thymoma. An advantage in terms of operative outcomes, post-operative pain, cosmetic results and hospital stay was observed if compared to the alternative approaches. The short-term oncologic outcome was excellent based on the high complete resection rate of the tumor.

## 1. Introduction

Thymoma, although rare, is the most common type of anterior mediastinal tumor in adults [1]. As part of a multidisciplinary approach, surgery is still the treatment of choice for this neoplasm and completeness of resection is the prognostic factor allowing the higher rate of cure [2]. However, the choice for the best surgical approach has always been a controversial issue.

The anatomical location of the thymus in the antero-superior mediastinum has for many years led to the belief that median sternotomy was the ideal surgical approach allowing a better exposure and higher chance of radical resection. This indication has been considered particularly appropriate when thymoma is associated with Myasthenia Gravis due to the need of removing all anterior mediastinal fatty tissue in order to eliminate potential sites of anti-acetylcholine receptor autoantibody production [3]. Thoracotomy has gained acceptance as a valid alternative, especially for tumors with prevalent protrusion on one side of the chest, although considered less adequate for the complete removal of fatty tissue in myasthenic patients [1,2].

In recent years, increasing interest in minimally invasive surgical procedures and improvement in technology and devices have led to the widespread adoption in this setting of Video-Assisted Thoracic Surgery (VATS) and more recently of the Robotic Assisted Thoracic Surgery (RATS) [4]. Over the last decade, increased availability of the robotic system has promoted a rapid affirmation of the latter approach based on related technical peculiarities allowing to overcome some of the limitations of VATS [5].

Based on improved skill and growing amount of clinical data in robotic assisted surgery for thymoma, an increasing number of comparative studies has appeared in the literature over the recent years assessing the “state of the art” of this technique, but only a few reports have analyzed the results of robotic interventions in comparison with those of all other main approaches (sternotomy, thoracotomy and VATS) [6].

The present study aims to evaluate results of the first 2-year experience with RATS Thymectomy for thymoma in a high-volume university hospital in the setting of a comparative analysis with homogeneous groups of patients undergoing surgery for thymoma through the other main surgical approaches (sternotomy, thoracotomy and VATS).

## 2. Materials and Methods

Between May 2021 and September 2023, 40 consecutive patients underwent RATS Thymectomy for resection with radical intent of stage I to limited stage III thymoma (limited infiltration of the pericardium, lung, mediastinal pleura) at the Division of Thoracic Surgery of the Sant’Andrea Hospital of the Sapienza University in Rome.

Tumors were classified according to the WHO histo-pathologic classification system of thymic epithelial tumors, 5th edition [7], and staged in accordance with the Masaoka–Koga clinicopathologic system [8].

Patients available for follow-up, with complete data about their tumor stage and histology, the possible presence of Myasthenia or other paraneoplastic syndromes, treatment and post-operative outcome were included. Patients undergoing robotic surgery for mediastinal tumor other than thymoma during the study period were excluded from the study.

All patients underwent routine preoperative investigations including blood tests and electrocardiogram; echocardiogram and stress/stimulus tests were associated in patients with previous cardiac disease or newly found electrocardiographic changes.

Patients with possible involvement of the lung parenchyma by the neoplasm and those with Myasthenia Gravis also underwent preoperative functional tests to assess respiratory performance (Pulmonary Function Test). Patients with an established diagnosis of Myasthenia Gravis also underwent neurological reevaluation and optimization of medical therapy before surgery.

**Figure 1:**
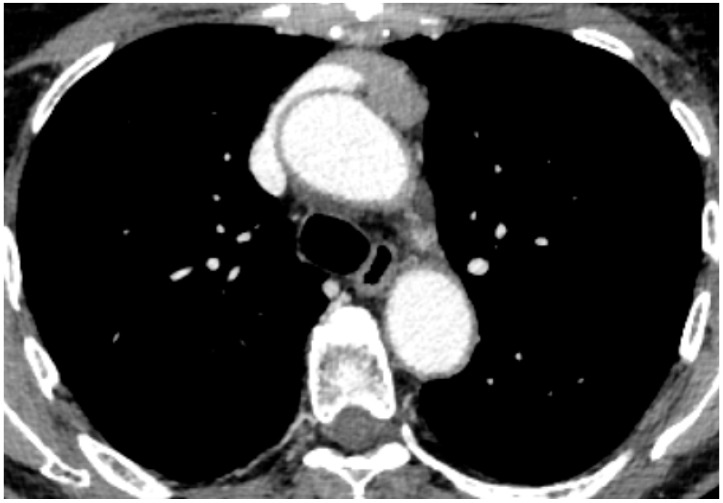
Preoperative CT scan image of thymoma undergoing RATS Thymectomy and thymomectomy with partial resection of the left innominate vein at CT scan.

The imaging study included total-body computed tomography (CT) with contrast medium (Figure 1) and Positron Emission Tomography with fluoro-desoxyglucose-18F (18FDG-PET) in all patients. Magnetic Resonance Imaging (MRI) of the brain was performed only in patients with specific contraindications to iodine contrast medium infusion [9].

The sternotomy approach was always a total median sternotomy; thoracotomy was performed by a 6 to 9 cm incision with a lateral sub-axillary muscle-sparing approach generally at the 4th intercostal space. VATS was generally performed by a bi-portal or tri-portal access. Location of surgical ports for VATS approach and use of utility access was chosen based on a case-by-case evaluation. RATS approach was generally standardized using three ports placed on the 5th intercostal space at midclavicular line and at the anterior axillary line and on the 3rd intercostal space at the middle axillary line.

Docking time is defined as the time spent to the anchorage of the robot to the patient and to the trocars’ insertion of robotic instruments into the chest cavity. The docking process consists of trocar insertion and positioning, driving the robotic arm to proximity of the patient, connecting the arms to the trocars and instrument insertion in the operating field. Undocking time is the time spent on robotic arm and trocar removal. The side of the video-thoracoscopic or robotic approach was chosen based on the prevalent protrusion of the neoplasm in the adjacent pleural cavities; in the case of equivalent tumor protrusion in both the pleural cavities, the left side was preferred. Surgeons who performed all the surgical procedures were a total of five. There were two first operating surgeons.

Data regarding the side of surgical approach, overall and actual operative time excluding docking-undocking procedures, associated adjacent structure resection, conversion rate, size of thymoma, histology, stage of disease, microscopically ascertained radicality (pR0), length of hospital stay, post-operative morbidity, mortality, pain, perceived cosmetic results and adjuvant oncologic treatment were recorded and investigated. Recurrence and mortality after the first month of the operation were also reported for the RATS group, but were not the object of comparative analysis with the other groups because of the short follow-up period.

Results concerning post-operative pain were obtained using the Numeric Rating Scale (NRS) from 0 (no pain) to 10 (maximum level of pain), based on patients’ subjective evaluation at 24 and 48 h. The cosmetic result at discharge and 1 month after surgery was also reported based on patients’ subjective evaluation using a scale from 0 (worst possible result and patient satisfaction) to 10 (best possible result and patient satisfaction) and compared. Comparison of results obtained in the RATS group was made with those of each group of patients undergoing sternotomy, thoracotomy or VATS. Last date of follow-up was 30 November 2024.

At the time of admission, informed consent was obtained from each patient for surgical treatment and for the use of clinical data anonymously for scientific disclosure purposes.

This retrospective study received the Institutional Review Board (Ethics Committee) approval (Cod. 5400/A0/22) of Ethics Committee University of Padua for National Multicenter research and Prot. No. C.8/2023 of Ethics Committee Sant’Andrea Hospital- Sapienza University of Rome) and it was conducted in accordance with the Declaration of Helsinki.

### 2.1. Statistical Analysis

Data were collected and categorized in Excel-type database (Microsoft Corporation, Redmund, WA, USA). Qualitative variables were expressed as mean or median ± standard deviation (SD), while nominal variables were expressed binary as presence (1) or absence (0) of the event.

Based on a mean amount of about 45 patients receiving surgery for thymoma in our institution per year, patients undergoing sternotomy, thoracotomy and VATS over the last 5 years were included for the comparison of results. To perform a comparative analysis, three additional groups consisting of 40 patients each, who had undergone thymectomy for radical removal of thymoma by other approaches (sternotomy, thoracotomy and VATS), were retrospectively identified using data from the institutional database, operative records, and pathology reports. Patients in the three groups were selected over a total of 172 patients (57 sternotomy, 52 thoracotomies, 62 VATS).

**Figure 2:**
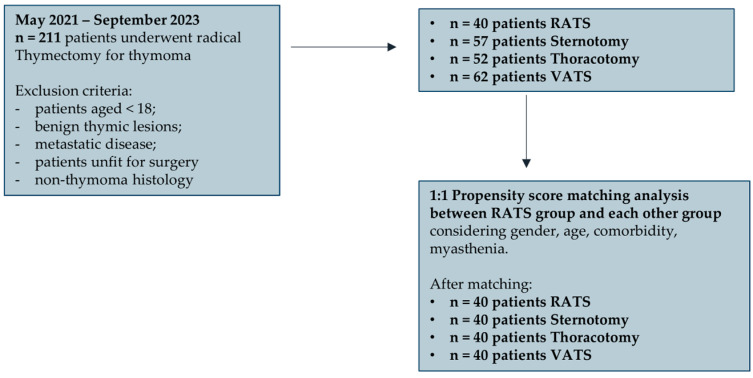
Propensity score matching analysis.

To minimize selection bias, a 1:1 propensity score matching was performed based on predetermined confounders and baseline characteristics (gender, age, comorbidity, Myasthenia and tumour stage) to identify homogenous groups of patients between RATS group and each other group: finally, 40 patients with homogenous characteristics were selected for each group (RATS, sternotomy, thoracotomy, VATS) (Figure 2).

### 2.2. Statistical Significance Is Expressed as a p < 0.05 Index

Comparative analysis of the data included comparison of the qualitative variables obtained in patients undergoing RATS in comparison with the other surgical approach techniques using a T test, while comparison of the nominal variables was performed using χ^2^ test or Fisher’s exact test.

All analyses were obtained by using the IBM SPSS Statistics statistical program version 25.0 (SPSS Software, IBM Corp., Armonk, NY, USA).

## 3. Results

Forty consecutive patients diagnosed with Masaoka stage I to III (limited-extension) thymoma underwent macroscopically complete surgical excision with thymectomy by robotic-assisted approach over a 29-month period (May 2021–September 2023). The mean age was 62.26 ± 8.57 years (range 41–81). There was a prevalence of females (n = 26, 65%). At the time of admission, eight patients (20%) were found to have Myasthenia Gravis, three in ocular form and five with bulbar and/or generalized symptoms.

All the RATS interventions were performed using the previously described standardized three ports approach to the antero-superior mediastinum. In all cases the approach was unilateral. The left approach was more frequently used (65%; n = 26).

The mean total operative time was 70.60 ± 8.13 min (range 70–220); mean operative time excluding docking-undocking procedures was 51.25 ± 16.52 min. Associated adjacent structures resections were performed in four (10%) patients. These included a right upper lobe lung wedge resection in two patients, tangential resection of the left Innominate Vein performed with vascular stapler at the confluence of Keynes veins in one and pericardial resection in one. There were no intraoperative events requiring conversion. All patients included in the study underwent surgery with curative intent and macroscopically complete excision (cR0). In a single patient, surgical resection was found to be not microscopically radical (R1) at final examination. Histopathologic analysis results and definitive pathologic staging according to Masaoka system are reported in Table 1.

If considering patients who underwent associated resection of surrounding structures, pathologic analysis confirmed the presence of stage III disease in only one patient, due to direct infiltration of the pericardium, while right lung parenchyma and vascular wall of the left innominate vein included in the specimen of the other three patients were found to be microscopically not infiltrated. The mean size (maximum diameter) of the thymoma was 6.14 ± 1.86 cm (range 4–10). The median post operative hospital stay length was 3 days and the mean was 3.36 ± 1.73. There was no operative mortality. The overall morbidity rate was 7.5%. Complications included moderate left pleural effusion requiring thoracentesis in one patient who underwent intervention through the right approach. In two other patients, recurrent episodes of paroxysmal atrial fibrillation occurred and resolved with pharmacological therapy. Pain subjectively reported by the patients according to NRS at 24 h and at 48 h had a mean score of 2.2 ± 0.8 and of 2.2 ± 0.7, respectively.

Subjectively perceived cosmetic results using a 0 to 10 scale was reported by patients with a mean score of 9.1 ± 0.5 (Table 2).

At a median follow-up of 24 months (range 15–42), no patients experienced local or systemic disease recurrence and there was no mortality.

The other three groups of 40 patients undergoing surgical treatment of thymoma through the alternative surgical approaches (sternotomy, thoracotomy, VATS) had homogeneous baseline characteristics, which are reported for each group in Table 1.

Mean overall operative time of RATS was significantly shorter compared to that of sternotomy (0.008) and VATS (0.03). It was similar to the operative time of thoracotomy interventions (*p* = 0.5), but resulted shorter when analyzing it without the docking-undocking procedures (*p* = 0.005) [Table 2].

The overall complication rate did not show significant difference among the four groups of patients; however, the major complication rate was lower in the RATS group (7.5%) compared to each of the other three groups (VATS, sternotomy, or thoracotomy), which presented all the same morbidity rate (12.5%). Type and distribution of complications in the four study groups are summarized in Table 3.

A higher prevalence of associated surrounding structures resections [Table 4] was observed in patients operated through sternotomy (35%; 14 patients; *p* = 0.015).

If considering patients undergoing minimally invasive surgery (VATS and RATS), the conversion rate was significantly higher (15%) during VATS compared with RATS (no conversion observed in this group; *p* = 0.026).

No significant difference in the distribution by stage and histologic type was observed in the four groups of the study (Table 1). Tumor size in patients receiving resection through sternotomy (mean tumor long axis of 7.72 ± 1.25 cm) was found to be slightly higher than that of tumor removed by RATS, although this difference was not statistically significant (*p* = 0.089). The mean tumor size reported in the group of patients who underwent thoracotomy and VATS was 6.21 ± 1.49 cm and 4.71 ± 0.48 cm, respectively, and did not show significant difference if compared with the mean tumor size in the RATS group.

The mean post-operative hospital stay duration in the group of patients undergoing robotic-assisted procedures was significantly shorter compared to the sternotomy group (3.36 ± 1.73 vs. 7.44 ± 3.67; *p* < 0. 001); the mean hospital stay after RATS was also shorter than that reported after thoracotomy, although without statistically significant difference (3.36 ± 1.73 vs. 4.12 ± 1.51; *p* = 0.104). There was no difference in mean post-operative hospital stay between RATS and VATS groups (3.36 ± 1.73 vs. 3.64 ± 1.38; *p* = 0.530).

Patients’ subjectively reported pain at 24 h and 48 h was significantly lower in the RATS group compared to the other approaches (*p* < 0.05) [Table 2].

Cosmetic results as reported by the patients were significantly better in the RATS group compared to the sternotomy group (9.1 ± 0.5 vs. 4.1 ± 0.8, *p* = 0.0001) and to the thoracotomy group (6.1 ± 0.9, *p* = 0.001). Cosmetic scores of the RATS group were also higher than those of the VATS group (8 ± 0.8) with this difference being at the limit of statistical significance (*p* = 0.052).

After surgery, four patients (10%) received adjuvant radiotherapy in the RATS group because of the presence of microscopic neoplastic foci at the resection margin in one patient or the presence of capsule infiltration associated with aggressive histology (B3 thymoma) in three other patients. In the sternotomy group, post-operative radiotherapy was administered in 15 patients (37.5%) with capsule infiltration and unfavorable histology (B2–B3). In the thoracotomy group, post-operative RT was administered in n = 8 patients (20%) with capsule infiltration and aggressive histology (B2-B3) and in 4 patients (10%) with incomplete microscopic excision (R1). In the VATS group, post-operative RT was administered in 11 patients (27.5%) because of capsule infiltration and more aggressive histology (B2–B3) and in 1 patient (2.5%) because of R1 resection. No patient received adjuvant chemotherapy.

## 4. Discussion

Surgery represents the treatment of choice for thymoma and radicality of resection has been proven to be the most significant favorable prognostic factor [10].

Median sternotomy has been the gold standard approach for these neoplasms for a long time [10,11]. Similarly, thoracotomy has been considered an adequate alternative for anterior mediastinal tumors with prevalent protrusion in the pleural cavity on one side. However, over the last decades minimally invasive surgery techniques, including VATS and more recently RATS, have gained increasing preference among surgeons and patients, thus becoming the standard of treatment for early stage thymoma. Several studies have recently appeared in the literature comparing the perioperative outcomes of minimally invasive approaches with those of sternotomy and thoracotomy and have shown adequate safety and efficacy [12], and comparable, if not superior, oncological outcomes [13].

Sternotomy is an invasive surgical procedure, which may lead to a higher risk of complications such as perioperative bleeding, prolonged post-operative inability, severe dehiscence and infection [14]. With the advent of minimally invasive approaches, VATS has become the most popular and commonly used option, resulting in reduction of perioperative major morbidity [15].

**Figure 3:**
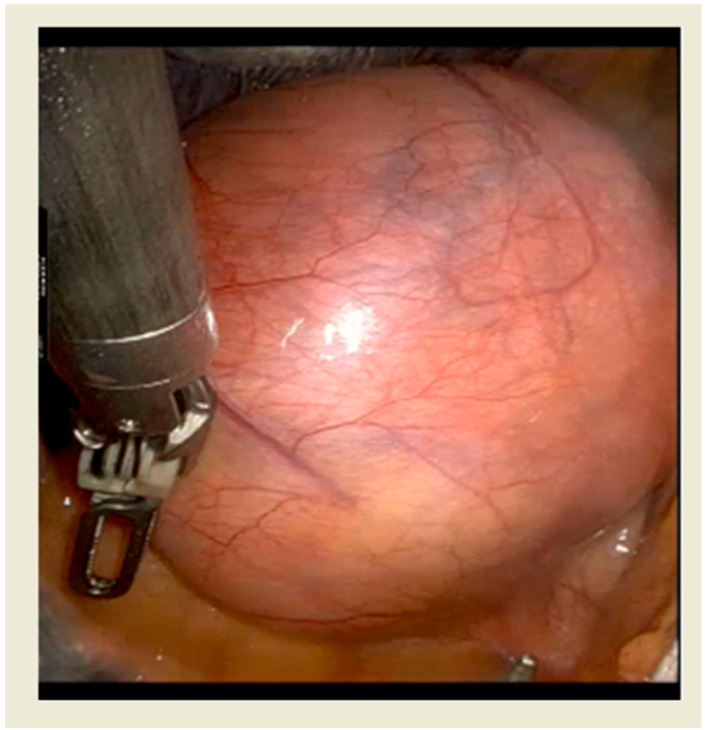
Intraoperative view of a 9-cm thymoma undergoing resection with RATS approach.

Robotic thymectomy represents the most recent minimally invasive surgical approach to the thymic gland and the anterior mediastinum showing several technical advantages, which allow to overcome some limitations of VATS. The Da Vinci System is the most used technology in this field with a well consolidated experience to date. Main technical advantages include high-quality three-dimensional vision with image magnification (Figure 3) and more accurate and precise movements of surgical instruments in limited space, especially due to 7 degrees of freedom and the tremor filtering system. This provides a more adequate visualization and manipulation of anatomical structures with easier identification of surgical planes, allowing a more effective dissection [16]. Therefore, experiences with this technique published in the literature have reported a significant improvement in results. Shen et al. [17] and Wu et al. [18] have compared the short-term outcome of RATS with that of VATS thymectomy by a meta-analysis. RATS resulted in reduction of intraoperative blood loss, shortening of post-operative drainage permanence, reduction of post-operative drainage volume, reduction of hospitalization time and of post-operative complication rate [19].

The main concern limiting the diffusion of robotic approach in the early era of this technique is related to the possibility of tumor capsule rupture and potential tumor cells implantation with higher risk of local recurrence. However, evidence from the literature results and from our experience have clearly demonstrated the operative and long-term oncological reliability of the RATS treatment based on high rates of R0 resection, no increase of recurrence rate and long-term survival comparable with that of open techniques [20].

In our present experience, we have compared the surgical results of patients undergoing RATS Thymectomy with those of other three groups of patients with similar baseline characteristics receiving the same operation by alternative approaches. The RATS group presented a significant improvement in terms of length of hospital stay compared to the sternotomy group and even shorter hospitalization when compared with the thoracotomy and VATS group, although without statistical significance. These data are in line with those of other published series representing the proof of improved efficacy of the robotic technique [21].

Operative time is obviously always influenced by the learning curve with longer surgery duration at the beginning of the RATS experience [22]. However, the present study, although analyzing the initial RATS experience performed in our institution, shows favorable results of operative time in comparison with those reported with the other three surgical approaches, especially if excluding the docking-undocking procedures. In particular, we reported significantly reduced mean RATS operation time compared to that reported with sternotomy and VATS, and with that of the thoracotomy group if excluding the docking-undocking time.

Thymic tumor size has been frequently considered as a factor to select patients for minimally invasive instead of open thymectomy, and several studies in the literature indicate the size of thymoma up to 4 cm as a safe threshold for a minimally invasive approach [23]. Kimura and colleagues [24] reported that VATS approach for thymomas larger than 5 cm increased the operative risk of capsular injury. Similarly, over the past decade, the RATS approach has been mainly offered to patients with limited thymoma size [25]. However, with increasing experience and technical skill, given the advantages provided by improved three-dimensional visualization of anatomical structures and instrument eclecticism, RATS has proved to allow safe resection of lesions of even more than 4 cm [26]. Comacchio et al., in an Italian nation-wide multicenter study, found that the median diameter of resected tumor was 4 cm, but in more experienced centers resection of tumors up to 16 cm were performed [23]. In our experience the mean tumor size in RATS group was 6.14 ± 1.86 cm (including resected thymoma up to 10 cm), without significant differences if compared with the other groups.

Moreover, in the study by Chiba et al., the RATS approach was found to be related with improved post-operative Quality of Life (QOL) in comparison with VATS, principally because of a shorter duration of chest tube which was associated with decreased pain [12]. In our experience, patients undergoing RATS intervention presented a significant pain reduction both at 24 h and 48 h compared to the other approaches (*p* < 0.05). Even cosmetic results were better in the RATS group, especially when compared to sternotomy (*p* = 0.0001) and thoracotomy (*p* = 0.001), thus improving patients’ satisfaction and perception of a better QoL [27].

To date, limited data are available on long-term oncologic outcomes after RATS. It is however recognized that R0 resection is the most significant surgical prognostic factor [28]. Comacchio et al. confirmed that a high rate of complete R0 resection (90%) can be achieved using robotic thymectomy for patients with large thymomas, and in a recent analysis of the International Thymic Malignancies Interest Group registry data, Burt and colleagues [29] observed that neither surgical approach nor tumor size correlated with R0 resection status.

In our experience, R0 was achieved in the 97.5% of patients in the RATS group, thus supporting the efficacy of RATS Thymectomy for oncological radicality.

The overall complication rate in our experience did not show significant difference among the four groups of patients; however, the major complication rate was lower in the RATS group (7.5%) compared to each of the other three groups (VATS, sternotomy or thoracotomy), which presented all the same morbidity rate (12.5%).

Although kidney injury is reported in a few series in the literature [30], this severe injury has not been described in our series.

General criteria for minimally invasive approach indication usually include tumor encapsulation and the presence of a cleavage plane with adjacent cardio-vascular structures. However, the recent multi-institutional Italian study [23] reported the need for associated resection of surrounding structures in 57 over a total of 669 patients (8.5%). Nevertheless, a low conversion rate (3.4%) was reported, which was in line with the main previous published data reporting this incidence between 1% and 10% [11,12,13,14,15,16,17,18,19,20,21,22,23,24,25,26,27,28,29,30,31]. These data prove that the need for extended resection, in case of surrounding structure limited infiltration, can be managed by robotic approach without conversion in most cases. In our experience, associated resection of adjacent structures was performed in 7.5% of patients of the RATS group, which was a lower rate if compared to the sternotomy group. However, we reported no need for conversion due to intraoperative complications or indication for extended resection.

The main limitations of the present study are represented by the inclusion of an initial experience results (which are obviously influenced by the learning curve), and by the retrospective, observational nature of the study. However, the strength is related to the homogeneity of the groups of patients considered for the comparative analysis, the short study period (limiting differences in patients management over time) and the adequateness of statistical sample if considering the single institution nature and the brief study period.

## 5. Conclusions

In conclusion, based on data obtained from our initial experience, RATS Thymectomy with thymomectomy has been confirmed to be a safe and effective technique for the treatment of thymoma up to Masaoka stage III disease with limited extension, allowing accurate and complete excision of the tumor. Operative outcomes and morbidity have been shown to be equivalent to or even better than those obtained with the most used alternative approaches. In particular, the RATS technique is associated with a significant reduction of operative time and significantly higher patient-perceived satisfaction in terms of cosmetic outcome and post-surgical pain compared to all other main techniques. Moreover, it is the technique showing the shortest mean hospital stay with a significant difference if compared with sternotomy. Short-term oncologic outcomes after robotic resection are also excellent and in line with those reported from major surgical series in the literature

## Figures and Tables

**Table 1:** General patient characteristics.

	RATS 40	Sternotomy 40		Thoracotomy 40		VATS 40	
	n (%)	n (%)	*p*	n (%)	*p*	n (%)	*p*
Age, mean ± SD	62.26 ± 8.57	54.14 ± 11.43	0.299	61.34 ± 9.8	0.892	61.28 ± 9.8	0.885
Gender (M/F)	14/26	16/24	0.817	10/30	0.465	15/25	1
Myasthenia	8 (20%)	16 (40%)	0.087	6 (15%)	0.769	7 (17.5%)	1
Morbidity	3 (7.5%)	5 (12.5%)	0.712	5 (12.5%)	0.712	5 (12.5%)	0.712
Histology							
A	5 (12.5%)	5 (12.5%)	1	11 (27.5%)	0.161	8 (20%)	0.546
AB	13 (32.5%)	10 (25%)	0.622	11 (27.5%)	0.161	14 (35%)	1
B1	5 (12.5%)	10 (25%)	0.252	10 (25%)	0.252	7 (17.5%)	0.755
B2	5 (12.5%)	10 (25%)	0.252	5 (12.5%)	1	8 (20%)	0.546
B3	12 (30%)	5 (12.5%)	0.099	3 (7.5%)	0.019	3 (7.5%)	0.019
Masaoka							
I	10 (25%)	13 (32.5%)	0.622	10 (25%)	1	16 (40%)	0.232
IIA	21 (52.5%)	13 (32.5%)	0.113	20 (50%)	1	11 (27.5%)	0.039
IIB	8 (20%)	11 (27.5%)	0.6	10 (25%)	0.789	13 (32.5%)	0.309
III	1 (2.5%)	3 (7.5%)	0.615	0 (0%)	1	0 (0%)	1

**Table 2:** Operative and post-operative results.

	RATS 40	Sternotomy 40		Thoracotomy 40		VATS 40	
	n (%)	n (%)	*p*	n (%)	*p*	n (%)	*p*
Surgery time, mean ± SD	70.60 ± 8.13	116.41 ± 22.48	0.008	76.25 ± 13.15	0.598	90 ± 11.55	0.030
Surgery time without docking, mean ± SD	51.25 ± 16.52	116.41 ± 22.48	0.003	76.25 ± 13.15	0.005	90 ± 11.55	0.008
Associated resections	4 (10%)	14 (35%)	0.015	3 (7.5%)	1	0 (0%)	0.116
Conversion rate	0 (0%)	0 (0%)	1	0 (0%)	1	6 (15%)	0.026
R0	39 (97.5%)	40 (100%)	1	36 (90%)	0.036	39 (97.5%)	1
Lenght of stay in days, mean ± SD	3.36 ± 1.73	7.44 ± 3.67	<0.001	4.12 ± 1.51	0.104	3.64 ± 1.38	0.530
Dimensions, mean ± SD	6.14 ± 1.86	7.72 ± 1.25	0.089	6.21 ± 1.49	0.877	4.71 ± 0.48	0.073
Pain, mean ± SD							
24 h	2.2 ± 0.8	5.2 ± 0.9	0.003	5.8 ± 0.7	0.001	4.3 ± 0.5	0.004
48 h	2.2 ± 0.7	5.1 ± 0.6	0.004	5.3 ± 0.7	0.001	4.0 ± 0.9	0.014
Cosmetic results, mean ± SD	9.1 ± 0.5	4.1 ± 0.8	0.0001	6.1 ± 0.9	0.001	8 ± 0.8	0.052

**Table 3:** Complications.

Approach	Type of Complications	n	N (%)
RATS	Atrial fibrillation	2	3 (7.5%)
	Pleural effusion	1	
Sternotomy	Phrenic nerve injury	2	5 (12.5%)
	Ventricular fibrillation	1	
	Bleeding	1	
	Pneumonia	1	
Thoracotomy	Thoracotomy dehiscence	1	5 (12.5%)
	Hemothorax (re-intervention)	2	
	Pneumonia	2	
VATS	Pulmonary embolism	1	5 (12.5%)
	Myasthenic crisis	1	
	Bleeding	2	
	Pneumonia	1	

**Table 4:** Associated adjacent structure resection.

Approach	Side	Type of Associated Resection	N; %
RATS	2 L 2 R	1 anonymous vein 2 lung (wedge upper lobe) 1 pericardium	4; 10%
Sternotomy	2 L, 3 R	5 pericardium	14; 35%
	4 L, 2 R	6 lung (wedge upper lobe)	
	2 L	2 phrenic nerve	
	1 R	1 pleura	
Thoracotomy	1 L	1 phrenic nerve	3; 7.5%
	1 L, 1R	2 lung (wedge upper lobe)	
VATS	0	0	0; 0%

L = left; R = right.

## Data Availability

All data are presented in the manuscript.
